# Endothelial superoxide dismutase 2 is decreased in sickle cell disease and regulates fibronectin processing

**DOI:** 10.1093/function/zqac005

**Published:** 2022-02-17

**Authors:** Atinuke Dosunmu-Ogunbi, Shuai Yuan, Daniel J Shiwarski, Joshua W Tashman, Michael Reynolds, Adam Feinberg, Enrico M Novelli, Sruti Shiva, Adam C Straub

**Affiliations:** Medical Scientist Training Program, University of Pittsburgh School of Medicine, 15261, Pittsburgh, PA, USA; Department of Pharmacology and Chemical Biology, University of Pittsburgh School of Medicine, 15261, Pittsburgh, PA, USA; Heart, Lung, Blood and Vascular Medicine Institute, University of Pittsburgh, 15261, Pittsburgh, PA, USA; Heart, Lung, Blood and Vascular Medicine Institute, University of Pittsburgh, 15261, Pittsburgh, PA, USA; Department of Biomedical Engineering, Carnegie Mellon University, 15261, Pittsburgh, PA, USA; Medical Scientist Training Program, University of Pittsburgh School of Medicine, 15261, Pittsburgh, PA, USA; Department of Biomedical Engineering, Carnegie Mellon University, 15261, Pittsburgh, PA, USA; Heart, Lung, Blood and Vascular Medicine Institute, University of Pittsburgh, 15261, Pittsburgh, PA, USA; Department of Biomedical Engineering, Carnegie Mellon University, 15261, Pittsburgh, PA, USA; Department of Materials Science and Engineering, Carnegie Mellon University, 15261, Pittsburgh, PA, USA; Department of Pharmacology and Chemical Biology, University of Pittsburgh School of Medicine, 15261, Pittsburgh, PA, USA; Heart, Lung, Blood and Vascular Medicine Institute, University of Pittsburgh, 15261, Pittsburgh, PA, USA; Department of Pharmacology and Chemical Biology, University of Pittsburgh School of Medicine, 15261, Pittsburgh, PA, USA; Heart, Lung, Blood and Vascular Medicine Institute, University of Pittsburgh, 15261, Pittsburgh, PA, USA; Department of Pharmacology and Chemical Biology, University of Pittsburgh School of Medicine, 15261, Pittsburgh, PA, USA; Heart, Lung, Blood and Vascular Medicine Institute, University of Pittsburgh, 15261, Pittsburgh, PA, USA; Center for Microvascular Research, Department of Medicine, University of Pittsburgh School of Medicine, 15261, Pittsburgh, PA, USA

**Keywords:** fibronectin, sickle cell, sod2, endothelium, redox, reactive oxygen species, mitochondria

## Abstract

Sickle cell disease (SCD) is a genetic red blood cell disorder characterized by increased reactive oxygen species (ROS) and a concordant reduction in antioxidant capacity in the endothelium. Superoxide dismutase 2 (SOD2) is a mitochondrial-localized enzyme that catalyzes the dismutation of superoxide to hydrogen peroxide. Decreased peripheral blood expression of SOD2 is correlated with increased hemolysis and cardiomyopathy in SCD. Here, we report for the first time that endothelial cells exhibit reduced SOD2 protein expression in the pulmonary endothelium of SCD patients. To investigate the impact of decreased SOD2 expression in the endothelium, SOD2 was knocked down in human pulmonary microvascular endothelial cells (hPMVECs). We found that SOD2 deficiency in hPMVECs results in endothelial cell dysfunction, including reduced cellular adhesion, diminished migration, integrin protein dysregulation, and disruption of permeability. Furthermore, we uncover that SOD2 mediates changes in endothelial cell function via processing of fibronectin through its inability to facilitate dimerization. These results demonstrate that endothelial cells are deficient in SOD2 expression in SCD patients and suggest a novel pathway for SOD2 in regulating fibronectin processing.

## Graphical Abstract

**Graphical Abstract:**
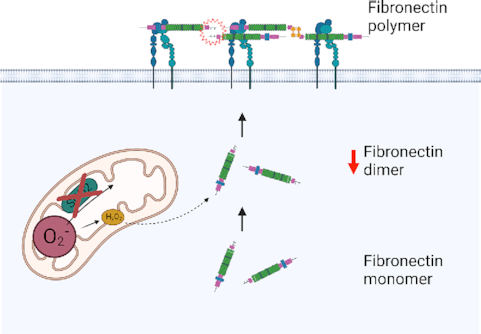
Assessment of fraction new over time reveals insight into proteostatic maintenance in muscle collagen.

## Introduction

Sickle cell disease (SCD) is an inherited hemoglobinopathy that results in sickling of deoxygenated red blood cells (RBC) due to a point mutation in the beta-globin gene.^1^ In SCD, there is an increase in oxidative stress due to augmented reactive oxygen species (ROS) production from enzymatic sources such as xanthine oxidase^2^ and non-enzymatic sources including Fenton chemistry.^3^ Paired with this increase in ROS production is a compromised antioxidant system. Levels of both enzymatic and non-enzymatic antioxidants are diminished in sickle cell patients compared to controls.^4–6^ This reduction in antioxidants leaves reactive species free to target a variety of molecules such as cysteines and lipids, leading to endothelial dysfunction, including sterile inflammation.^7^ As such, activated endothelium upregulates surface adhesion molecules causing RBC adhesion and microvascular occlusion.^7^ Extensive microvascular occlusions contribute to end-organ damage, which in SCD may manifest as acute chest syndrome,^8^ pulmonary hypertension,^9–11^ stroke,^12^, ^13^ acute splenic sequestration,^14^ papillary necrosis,^15^, ^16^ amongst others determined by the organ affected.^17^ Pulmonary endothelial dysfunction is responsible for pulmonary hypertension, which significantly escalates mortality of SCD patients and is thus a cause of clinical concern.^9^, ^18^, ^19^ Both in SCD and other conditions, pulmonary hypertension is characterized by increased oxidative stress^20^ and, in severe cases, a decrease in the enzymatic antioxidant superoxide dismutase 2 (SOD2).^21^

Superoxide dismutase 2 is a mitochondrial-localized enzymatic antioxidant that functions by dismutating superoxide to hydrogen peroxide (H_2_O_2_).^22^ SOD2 has been shown to play an essential role in maintaining vascular function, while SOD2 deficiency increases oxidative damage and disrupts vascular homeostasis.^22^, ^23^ *In vivo*, depletion of SOD2 promotes neointimal formation through the attenuation of vascular smooth muscle cell (SMC) proliferation and migration.^24^ Additionally, cardiomyocyte depletion of SOD2 in a mouse model results in the development of a dilated cardiomyopathy with isolated cardiomyocytes producing more ROS resulting in a disruption in oxidative phosphorylation.^25^ However, the role of SOD2 in endothelial cells remains to be investigated.

In SCD, there is reduced peripheral blood expression of SOD2 and this decrease in SOD2 is associated with increased hemolysis, inflammation, iron overload, oxidative stress, and SCD cardiomyopathy.^26^ While reduction of peripheral blood SOD2 has been reported, the study was limited due to a small sample size and select patient population. Consequently, there has been little investigation into the consequences of reduced SOD2 in SCD. More importantly, it is not clear whether vascular endothelial SOD2 is affected in SCD patients. Considering that the endothelium is a primary target for oxidative stress in SCD and that SOD2 is decreased in pulmonary vascular disease, we hypothesized that endothelial cell expression of SOD2 would be reduced in SCD lungs and that suppression of SOD2 protein levels in cultured endothelial cells would lead to mitochondria dysfunction and subsequent endothelial cell dysfunction.

## Materials and Methods

### Human Lung Tissue Samples

Human lung samples were explanted from four patients with SCD and no history of lung disease. SCD patient samples were obtained through Institutional Review Board (IRB) approved protocols at the University of Pennsylvania.^27^ Lung samples were formalin fixed and paraffin embedded then cut into 4- to 5-μm thick sections. Sections were then used for immunofluorescent staining. Patients were 44.2 ± 9.9 years old at the time of death and two were female. Samples from four African American controls were obtained under University of Pittsburgh CORID #451. Controls were 43 ± 13.7 years, two were female.

### Immunofluorescent Staining

Tissue Histology: Lung sections were deparaffinized with xylene followed by rehydration by decreasing 100%–70% ethanol washes ending with distilled water. Sections underwent antigen retrieval in a heat mediated citric acid-based solution (H-3300, Vector Laboratories) for 20 min and then cooled at room temperature for 1 h. Sections were then blocked in 10% horse serum (H1270, Millipore Sigma) in PBS for 1 h at room temperature. Primary antibodies for von Willebrand Factor (ab11713, Abcam, 1:100) and SOD2 (ab13533, Abcam, 1:250) were diluted in 10% horse serum in PBS and incubated on sections at 4°C overnight. After primary antibody incubation, cells were washed in PBS and incubated with secondary antibodies donkey anti-rabbit Alexa Fluor 596 (A21207, Invitrogen, 1:250), and donkey anti-sheep Alexa Fluor 647 (A21448, Invitrogen, 1:250), along with preconjugated smooth muscle α-actin-FITC (ACTA2-FITC; F3777, MilliporeSigma, clone 1A4, 1:500) and DAPI (D3571, Thermo Fisher Scientific, 1:250) for 1 h at room temperature. Sections were then washed with PBS and a coverslip was placed on after adding Prolong Gold Antifade with DAPI reagent (P36931, Invitrogen) to each section. Lungs sections were imaged using a Nikon A1 Confocal Laser microscope at 40x magnification with 1096×1096 resolution at the Center for Biologic Imaging at the University of Pittsburgh. Z-stacks in 0.5μm increments were taken. Images in the figures are representative images of the maximum-intensity projections of the Z-stacks. To quantify levels of SOD2 in the endothelial cell and SMC layers, regions of interest were drawn from the maximum intensity projection of the von Willebrand factor (endothelial cells) and ACTA2 (SMCs) and superimposed onto the maximum-intensity projection for SOD2. Raw integrated intensity per area in ImageJ was then quantified to represent the amount of SOD2 staining in the endothelial and SMC layers.

Cell Histology: hPMVECs were grown on glass coverslips, fixed with 2%PFA for 30 min, washed with PBS and then permeabilized in 0.1% Trypsin for 20 min. Following permeabilization, cells were blocked in 1% BSA in PBS/FBS for 1 hour and then primary antibody for fibronectin (E5H6X, rabbit, Cell Signaling, 1:800) was diluted in 1% BSA in PBS/FBS overnight at 4°C overnight. After primary antibody incubation, cells were washed and incubated in donkey anti-rabbit Alexa Fluor 596 (A21207, Invitrogen, 1:250) and DAPI (D3571, Thermo Fisher Scientific, 1:250) for 1 h at room temperature. Sections were then washed with PBS and mounted with Prolong Gold Antifade with DAPI reagent (P36931, Invitrogen). hPMVECs were imaged using a Nikon A1 Confocal Laser microscope at 60x magnification with 4x zoom and 1096 × 1096 resolution at the Center for Biologic Imaging at the University of Pittsburgh. Z-stacks in 0.5 μm increments were taken. Images in the figures are representative images of the maximum-intensity projections of the Z-stacks.

### Mouse Strain and Collection of Lung Specimens

All animal studies were done under a protocol approved by the Institutional Animal Care and Use Committee of the University of Pittsburgh. Homozygous male sickle and non-sickle control Townes mice were obtained from The Jackson Laboratories (Bar Harbor, ME, stock No. 013071). Mice were aged 3 mo before sacrificing and collection of lung tissue. Lung tissue was flash frozen in liquid nitrogen, then ground using mortar and pestle before extracting protein and RNA for subsequent analysis. Mouse tissue was lysed in ice-cold 1x RIPA lysis supplemented with additional protease and phosphatase inhibitors (Sigma-Aldrich). Protein lysate concentrations were quantified using a BCA Protein Assay (23225, Thermo Fisher). Tissue was lysed in TRIzol reagent (15596026, Thermo Fisher). RNA from lysates were isolated according to the protocol from the Direct-zol RNA miniprep plus kit (R2051, Zymo).

### Cell Line, Tissue Culturing, and SOD2 Knockdown

Human pulmonary microvascular endothelial cells (hPMVECs, CC-2527, Lonza) were cultured at 37°C in Lonza Endothelial Cell Growth Medium-2 (CC-3156) with EGM^TM^-2 MV Microvascular Endothelial Cell Growth Medium-2 BulletKit^TM^ (CC-4147, Lonza) supplementation. Cells were passaged using Gibco Trypsin-EDTA (T4049, Sigma-Aldrich) up to passage 12. To induce gene knockdown of SOD2, hPMVECs were transiently infected with SOD2 siRNA (Dharmacon) using Lipofectamine 3000 (L3000001, Thermo Fisher) transfection reagent according to the manufacturer's protocol for 48–72 h. Control cells were similarly transfected with a non-targeting siRNA sequence (Dharmacon). 100 U/mL PEG Catalase was added overnight to select cells for treatment.

### Western Blotting

hPMVECs were cultured in twelve-well dishes until confluent and lysed in Laemmli sample buffer containing 1% SDS, 10% glycerol, 31.25 nM Tris-HCl, 0.005% Bromophenol Blue, and 2.5% β-Mercatoethanol. Lysates were boiled and run on 4%–12% BisTris polyacrylamide gels (Life Technologies) and transferred onto nitrocellulose membranes. Membranes were incubated in primary antibodies (Supplemental Table S1) at 4°C overnight. Secondary antibodies (Supplemental Table S1) were incubated for 1 h at room temperature. Visualization and analyses were completed with a LI-COR Odyssey Imager and Image Studio Software.

### Quantitative Reverse Transcription PCR

hPMVECs were cultured in twelve-well dishes until confluent and lysed in TRIzol reagent (15596026, Thermo Fisher). RNA from lysates were isolated according to the protocol from the Direct-zol RNA miniprep plus kit (R2051, Zymo). Isolated RNA was reverse transcribed to cDNA with SuperScript™ IV VILO™ Master Mix (11756050, Thermo Fisher). Quantitative PCR was performed using PowerUp SyBr Green Master Mix (A46012, Thermo Fisher) and 1 μM target primer (Supplemental Table S2) in a QuantStudio 5 Real-Time PCR System (Thermo Fisher).

### Mitochondria Superoxide Measurements

Transfected hPMVECs were tryspinized and then resuspended in Mg^2^ and Ca^2+^ free Hank's Balanced Salt Solution (HBSS). In a 96 well plate 150 000 cells and 5 μM MitoSOX Red™ (Invitrogen) were added. Fluorescent signal (510nm/580nm) was then recorded at 37°C for 2 h. In order to reduce variation, fluorescent intensities were averaged every 6 min before log phase slope was calculated to represent production rate. Results were normalized to protein concentration.

### Respiratory Complex Activities

Respiratory complex activity assays were performed as previously described.^28^ Complex activities were normalized to ATP citrate synthase activity.

### Measurement of Oxygen Consumption Rate

Oxygen consumption rate (OCR) measurements in hPMVECs were measured using the Seahorse Extracellular Flux (XF96) Analyzer (Seahorse Bioscience, Inc., North Billercia, MA). hPMVECs were seeded and grown overnight at a density of 2.2 × 10^4^ cells/well in standard XF24 plates. Next, growth media was replaced with DMEM and hPMVECs were consecutively treated with 2.0 μM oligomycin, 0.5 μM carbonyl cyanide-ρ-trifluoromethoxphenylhydrazone (FCCP), and 2.0 μM Rotenone/ 2.0 μM Antimycin A. Three measurements of OCR were made over 1.5 min after addition of the agents. OCR readings were normalized to cell number after crystal violet staining.

### ATP Measurements

CellTiter-Glo® Luminescent Cell Viability Assay (Promega, G9241) was used to quantify ATP according to manufacturer's instructions.

### Measurement of Δψ

Transfected hPMVECs were incubated with 10 μg/mL JC-1 probe (ThermoFisher Scientific, T3168) for 20 min at 37°C then subjected to 2 color flow cytometry (514/529 nm and 585/590 nm). The ratio of red aggregates to green monomers was calculated and expressed relative to siNT transfected hPMVECs.

### Transwell Assay

hPMVECs were grown to confluency on Transwell inserts (3450, Costar) containing 0.4 uM pores. About 1 mg/mL of albumin-fluorescein isothiocyanate (66 kDa) (A9771, Sigma) was added to the media over the cells. After 30 min, 1, 2, and 4 h 100 µL of media was extracted from below the Transwell insert and replaced with growth media. Extracted media was measured at 495 nm excitation and 519 nm emission peak wavelengths to determine endothelial cell monolayer permeability.

### Electric Cell Impedance Sensing

hPMVECs were plated at 60000/cm^2^ into 8-well polyethylene terephthalate arrays (8W10E+, Applied Biophysics). Measurements of transendothelial electric resistance (TEER) were obtained using an electric cell impedance sensing (ECIS) system (Applied Biophysics). Data was continuously collected every 63 s and recorded by computer and a Capacitance below 10 nF at 64 000 Hz was required for hPMVECs to be considered confluent. Baseline TEER was recorded for 1 h and cells were serum starved for 4 h before challenging hPMVECs with hydrogen peroxide or hemin.

### *In vitro* Wound Healing Assay

hPMVECs were transfected and grown to confluence in a 12 well tissue culture plate. Using a pipet tip two scratches were made within the well and an image was taken. The cells were incubated in growth factor free media for approximately 16 h and then the scratches were reimaged. ImageJ was used to measure the area of the scratch at 0 h and 16 h and percent wound closure was calculated. Four measurements were made from each well and three wells were plated for each condition. Figure represents average wound closures from experiments conducted on four separate days.

### Adhesion Assay

hPMVECs were transfected and grown to confluence in a six well cell culture plate. About 2 mM ethylenediaminetetraacetic acid (EDTA) was used to remove the cells from the plate and then resuspended in serum free media. Approximately 8000 cells were plated into each well of a 96 well plate. Two 96 well plates were simultaneously plated with cells and incubated at 37°C. After 20 min, one plate was removed from incubation, washed twice with PBS, fixed in 4% PFA, and then stained with 0.05% crystal violet. Wells were imaged using a light microscope and cells were counted. After 24 h, the second 96 well plate was removed from incubation and cells were fixed in 4% PFA, stained with 0.05% crystal violet, and washed with deionized water. Fixed and stained cells were then dissolved with 0.1% SDS and absorbance was read at 610 nm. Cell number was normalized to absorbance of wells from the 24-h plate. Five wells were plated for each condition in both the 20 min and 24 h 96 well plate. Figure represents average cell number from experiments conducted on three separate days normalized to the siNT cell number. To coat plates in fibronectin, 1 mg/mL fibronectin was added to each well enough to cover the surface and incubated at 37°C overnight. On the day of the experiment, fibronectin was removed from the plate and the plate was allowed to dry completely.

### Fibronectin Image Analysis

Fibronectin siNT and siSOD2 3D image stacks were analyzed using the FIJI implementation of ImageJ (NIH).^29^ The fibronectin image stacks were loaded into ImageJ and a set of custom macros were executed. The first macro maximally projected the stack in Z then segmented the fibronectin signal utilizing Phansalkar auto local thresholding. Bright pixel outliers were removed to decrease noise and the images were rotated to horizontally align the fibronectin band. The image was cropped to isolate analysis to the region containing the patterned fibronectin band of interest. Next, the image was made binary and the segmented particles were analyzed using the inbuilt particle analysis tool with circularity and other measures calculated automatically. The individual particle data and summary data were exported to Excel files. For 3D fibronectin analysis, surface objects were created in Imaris (Bitplane, v 9.5) analysis software for both the nucleus and the fibronectin channel. To minimize variability in the analysis across images and achieve consistent segmentation, the local contrast method was used for surface creation with a minimum value set to 750 for all images. The fibronectin surface was color coded using a statistic for sphericity. Image snapshots were captured and surface statistics were exported to Excel files for 3D visualization and quantification. Statistical analysis of particle data was then performed using GraphPad Prism 9 (GraphPad).

### Patterning Fibronectin Lines

The fibronectin lines were fabricated using an adaptation the surface-initiated assembly technique.^30^, ^31^ Briefly, 20 µm lines with 20 µm were first designed using AutoCAD software. The CAD file was then transferred to a transparency photomask (CAD/Art Services, Inc., Bandon, OR, USA), where the spaces and the segments of the lines were dark and transparent, respectively. Square glass wafers (Fisher No. 12-543-F 45  ×  50  ×  2  mm) were spin-coated with Photoresist SPR-220.3 at 5000  rpm for 20  s, baked on a 115 °C hot plate for 90  s, exposed to ultraviolet (UV) light through the transparency photomask, baked on a 115 °C hot plate for 90  s, and developed for 1 min using MF-26A developer. Sylgard 184 (Dow Corning) polydimethylsiloxane (PDMS) elastomer was prepared per manufacturer's directions by mixing 10 parts base to 1 part curing agent (by weight) using a Thinky-Conditioning mixer (Phoenix Equipment Inc., Rochester, NY, USA) for 2  min at 2000  rpm followed by 2  min of defoaming at 2000 rpm. PDMS was cast over the patterned photoresist-coated glass wafer and placed at 65 °C to cure the PDMS overnight. Square PDMS stamps ∼1 cm^2^ were cut out of the ∼5 mm thick PDMS layer.

Prior to stamping, 25 mm circular glass coverslips (Fisher No. 12-545-86 25CIR-1D) coverslips were cleaned, spin coated with PDMS, cured overnight, and functionalized using UV/Ozone for 15 min. The PDMS stamps were sonicated in 50% ethanol for 30 min, dried with nitrogen gas, and coated with a 50 μg/mL human FN solution (Corning). After 1 h of incubation at room temperature, the FN-coated PDMS stamps were rinsed in sterile ddH_2_O, dried with nitrogen, and stamped onto the PDMS coated coverslips. After 30 min, the PDMS stamps were carefully removed from the glass coverslips, leaving behind a microcontact-printed FN lines on the PDMS surface. Incubation with 1% Pluronic F-127 was performed for 15 min to prevent cells from adhering to the non-patterned PDMS. Cells were then seeded onto the coverslips following previously described methods.

### Statistics

Student's *t* test was used to determine significance using GraphPad Prism version 7.03 software.

## Results

### Patients With SCD Have Decreased SOD2 Expression in Pulmonary Endothelial and SMCs

**Figure 1.**
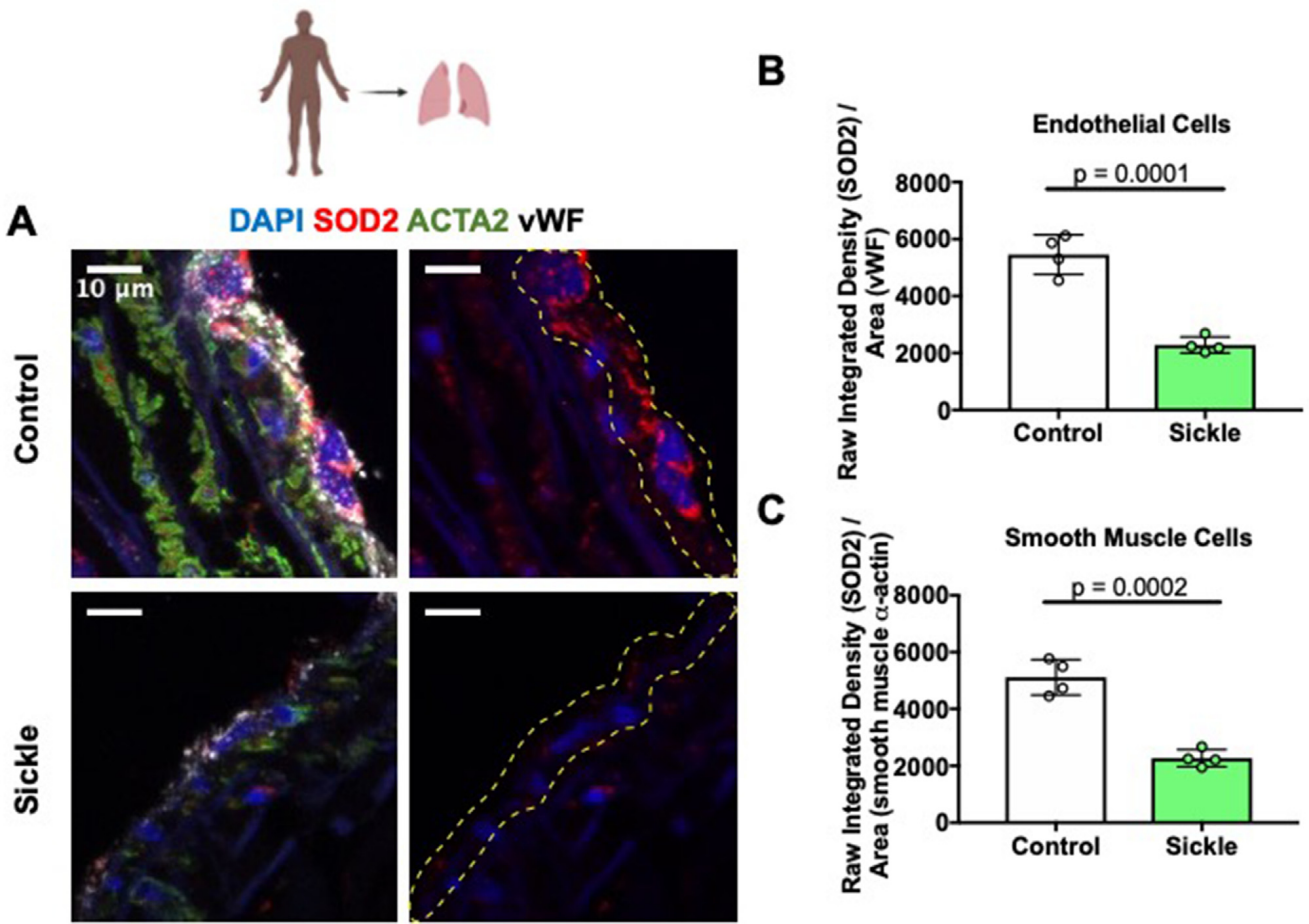
Patients with SCD have a reduction in endothelial and smooth muscle SOD2 protein expression. (A) SOD2 expression was measured by immunofluorescence in lung sections from 4 patients with SCD and 4 controls without overt lung disease. Expression of SOD2 (red; SMC stained green, von Willebrand factor stained white and DAPI stained blue) was increased in SCD patients compared to controls. (B, C) Quantification of endothelial cell or SMC SOD2 protein expression. 3-4 regions of interests were quantified for each patient and control sample and an average was taken and graphed. Values are means ± SD. *P*-values determined by two tailed Student's *t* test. Dotted line represents endothelial cell layer.

To determine SOD2 protein expression levels in the endothelium of patients with SCD, lung tissue samples from 4 patients (age 44.2 ± 9.9 years, 2 female) and 4 race matched controls (43 ± 13.7 years, 2 female) were stained with SOD2 (Figure 1A). Immunofluorescent staining analysis showed an approximately 40% reduction in SOD2 expression in SCD patient endothelial cells compared to controls (2283 ± 88.85 and 5518 ± 401.3, respectively) (Figure 1B). SOD2 staining in SMC was also decreased by roughly 40% in SCD patients compared to controls (2249 ± 86.84 and 5088 ± 405.5, respectively) (Figure 1C). This is the first known finding demonstrating a decrease in SOD2 protein expression in the pulmonary endothelial cells and SMCs of patients.

### Townes Transgenic Sickle Mice Have Dysregulation of Antioxidant Protein Expression in Whole Lung Tissue

**Figure 2.**
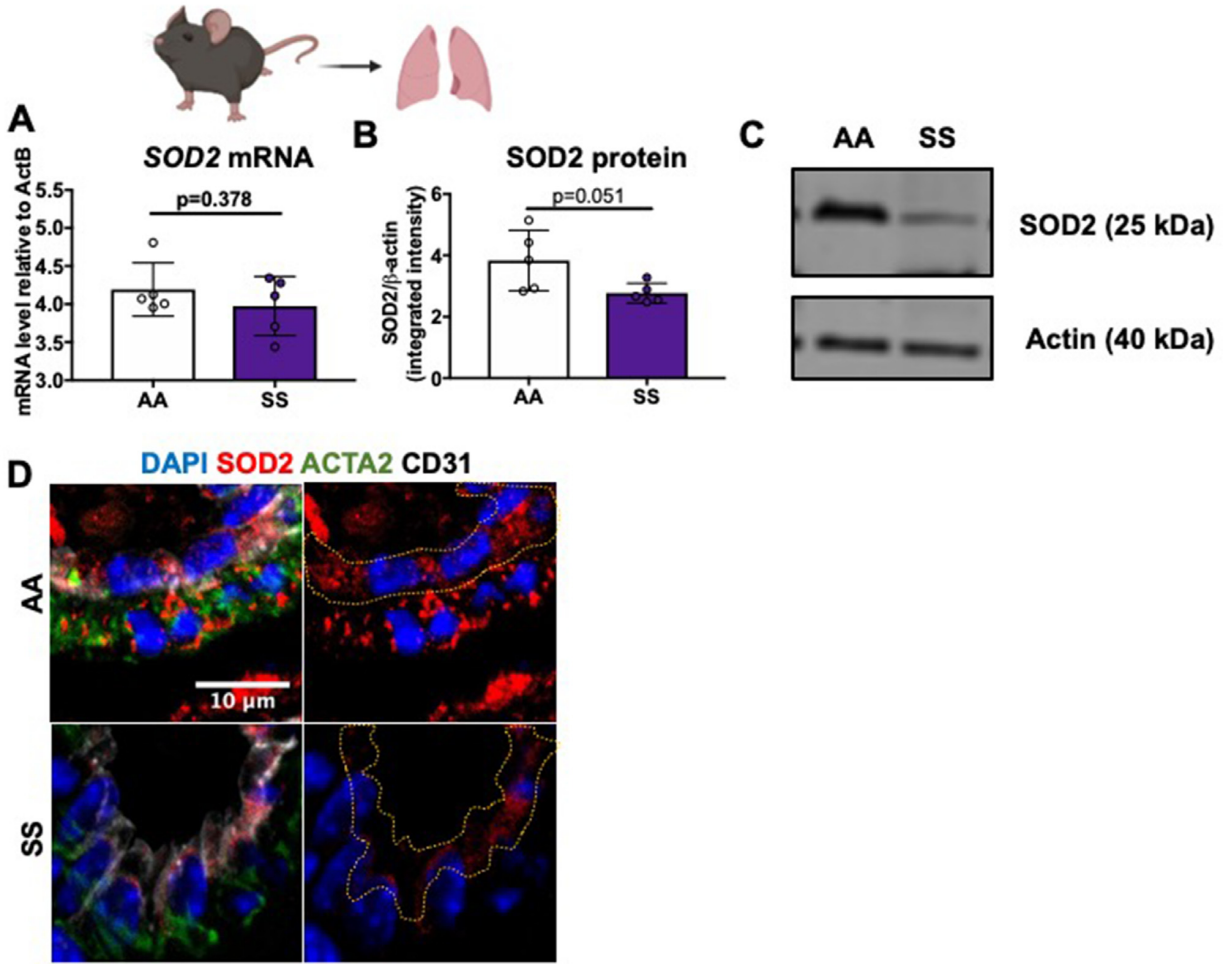
Townes transgenic SS mice compared to AA mice have decreased SOD2 protein expression in whole lung lysate. (A-C) Whole lung tissue of SS Townes mice (n = 5) had no changes in mRNA, but decreased SOD2 protein expression compared to AA Townes mice (n = 5). Quantification of SOD2 band density/actin band density from western blot. (D) SOD2 expression staining of lung sections from AA and SS Townes mice. SOD2 red; SMC stained green, von Willebrand factor stained white and DAPI stained blue. Data shown are individual values with means ± SD. *P*-values determined by two tailed Student's *t* test.

Next, SOD2 protein expression in the Townes transgenic sickle mouse model was investigated. mRNA and protein levels of all three SOD enzymes, as well as catalase in whole lung tissue of 3-mo-old sickle (SS) and non-sickle (AA) mice, were measured. While there was no change in *sod2* mRNA expression levels (Figure 2A), there was an approximately 25% decrease in SOD2 protein expression in SS mice as compared to age and gender-matched AA controls (1.914 ± 0.3504 and 3.833 ± 0.4401, respectively) (Figure 2B–D). In contrast, there were no changes in either *sod1* or *sod3* mRNA or protein expression (Supplemental Figure S1). There was, however, a decrease in *catalase* mRNA and an increase in protein expression of catalase (Supplemental Figure S1). These results, in conjunction with the SCD patient data, demonstrate SOD2 protein expression is decreased as a result of sickle pathology.

### Knockdown of SOD2 in hPMVECs Causes Mitochondrial Dysfunction

**Figure 3.**
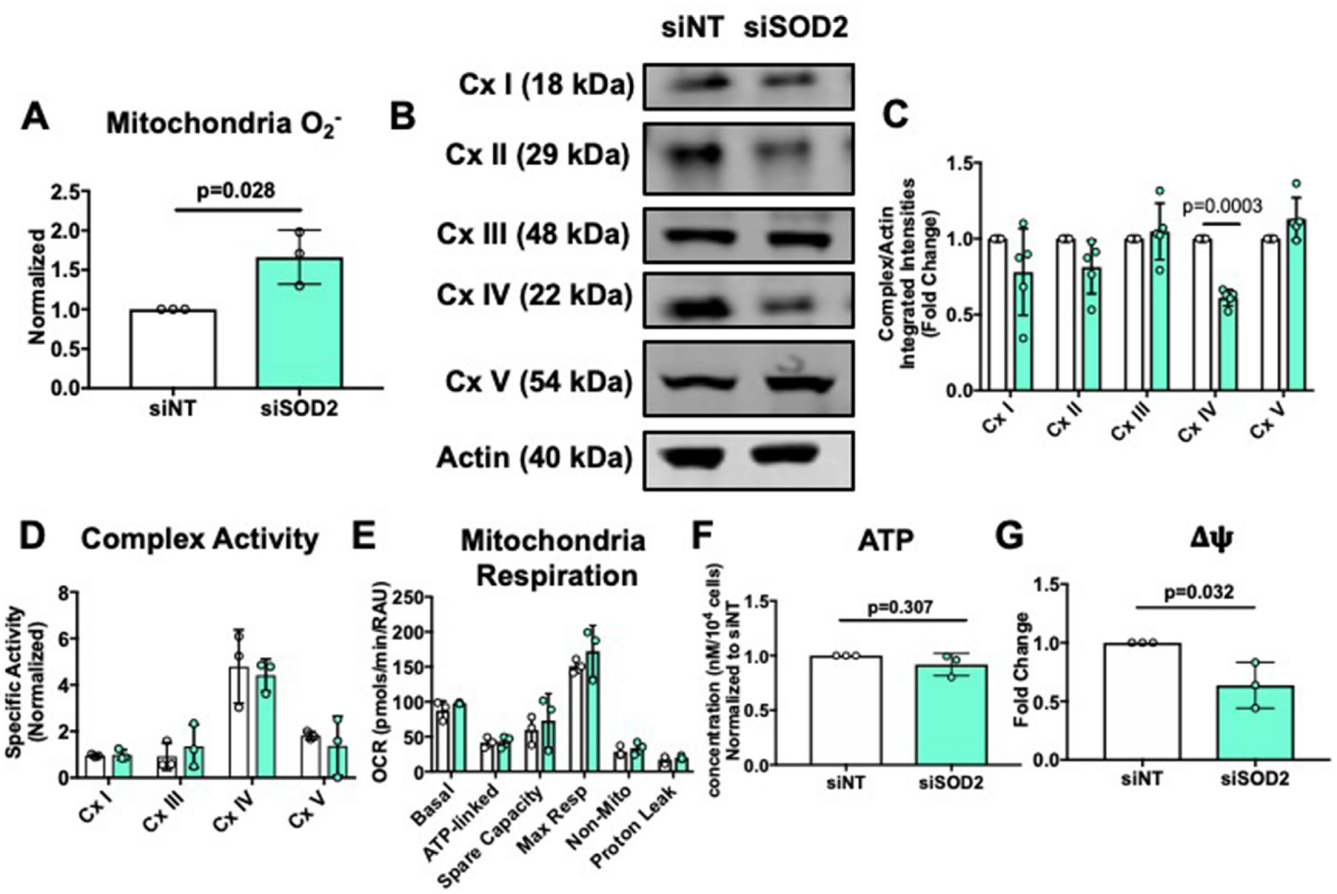
siSOD2 hPMVECs have increased mitochondrial superoxide production and decreased mitochondrial membrane potential. hPMVECs were transfected with non-targeting siRNA (siNT) or SOD2 siRNA to knockdown *sod2* mRNA and protein expression. Following 48 hours, experiments were conducted. (A) Mitochondria superoxide production in siNT and siSOD2 hPMVECs (n = 3). (B) Cells were lysed and Western blotted for mitochondria respiratory complex proteins. (C) Quantification of Western blot (n = 5). (D) Respiratory chain complex specific activity normalized to ATP citrate synthase activity in siNT and siSOD2 hPMVECs (n = 3). (E) Mitochondria oxidative consumption (n = 3), (F) Total ATP (n = 3) and (G) Mitochondria potential (n = 3) in siNT and siSOD2 hPMVECs. Data shown represent averaged values for each day of experimentation with means ± SD. *P*-values determined by two-tailed Student's *t* test.

To further determine the role of SOD2 in the pulmonary endothelium, siRNA was used to knockdown SOD2 in hPMVECs. While SOD2 siRNA decreased both *sod2* mRNA and SOD2 protein expression, there were no compensatory changes in *catalase* mRNA or catalase protein expression, though there was a significant decrease in SOD1 protein expression (Supplementary Figure S2). Next, to determine whether SOD2 knockdown affected ROS production, mitochondrial superoxide was measured and was found to be markedly increased in the absence of SOD2 (Figure 3A). We next tested mitochondria complex expression and function using a Seahorse extracellular flux technology. Mitochondria respiratory complex protein expression was first quantified, and we found that SOD2 knockdown resulted in a decrease in respiratory complex IV protein expression (Figure 3B and C). However, reduced complex IV protein expression did not affect complex specific activity, mitochondria respiration, or ATP production (Figure 3D–F), although the lack of SOD2 significantly decreased mitochondrial potential, indicative of damaged mitochondria (Figure 3G). Taken together, these data show that SOD2 deficiency in hPMVECs causes an amplified ROS production, decreasing mitochondria membrane potential but sparing mitochondria respiratory capacity.

### SOD2 Deficiency Increases Endothelial Permeability and Inhibits Migration and Proliferation

**Figure 4.**
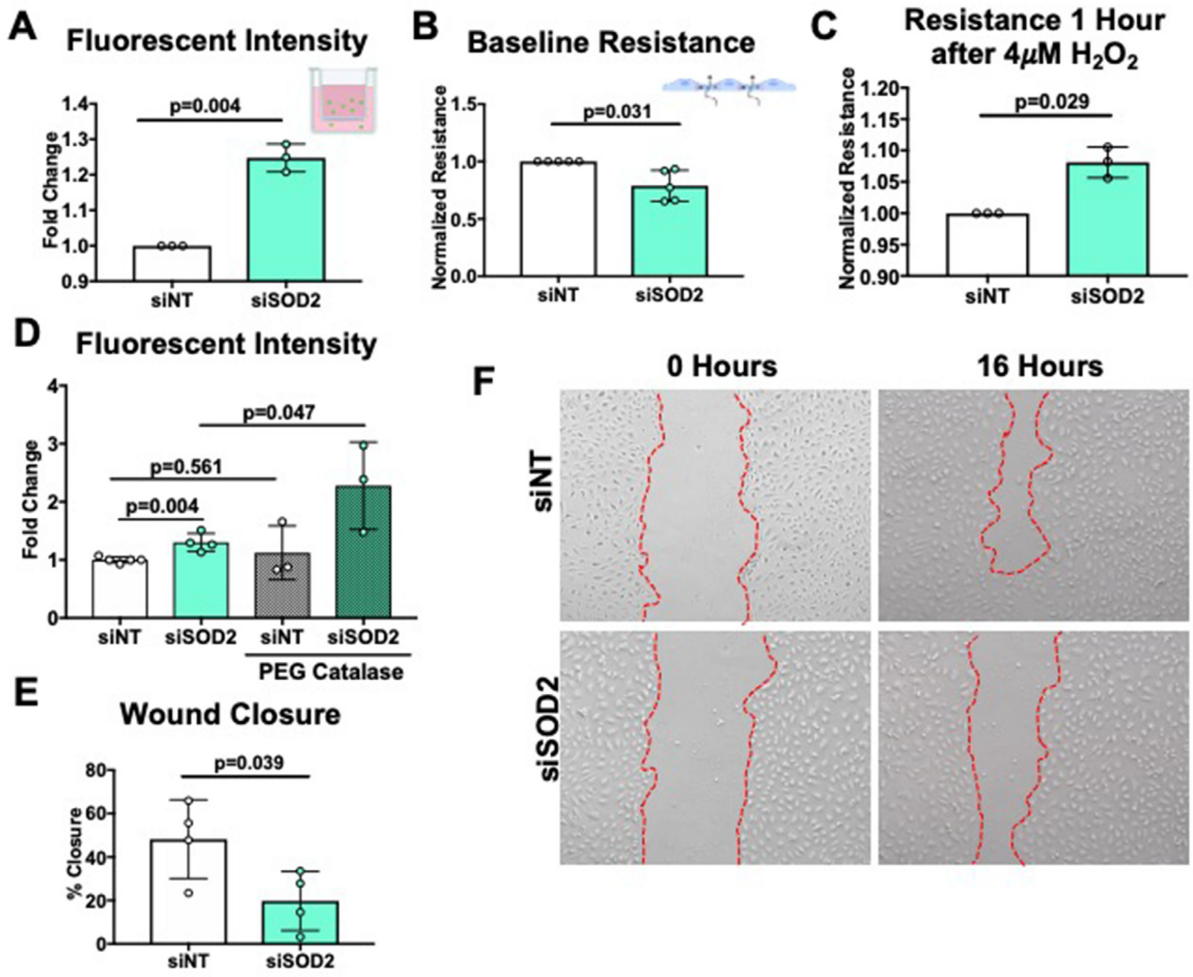
siSOD2 hPMVECs demonstrate increased permeability and decreased proliferation and migration. (A) siNT or siSOD2 hPMVECs were grown to a confluent monolayer on transwell filters. Albumin-fluorescein isothiocyanate was added to each well and medium from beneath the filter was collected at 1 h and fluorescent intensity was measured (n = 3). (B) siNT or siSOD2 cells were grown to a confluent monolayer on ECIS chips and baseline resistance was measured for 1 h (n = 5). (C) After serum starvation hPMVECs were treated with 4 μM H_2_O_2_ while measuring resistance (n = 3). (D) Fluorescent intensity from siNT and siSOD2 hPMVECs with and without 100 U/mL PEG catalase overnight treatment (E, F) Images and quantification of siNT and siSOD2 hPMVECs wound healing assay. Images were taken 0 and 16 h after wounding (n = 4). Data shown represent averaged values for each day of experimentation with means ± SD. *P*-values determined by two-tailed Student's *t* test.

A primary endothelial function is to serve as a barrier to confine blood from the interstitial space. To determine whether SOD2 regulates endothelial barrier integrity, hPMVECs were plated onto porous transwell inserts, and albumin leakage was measured over time. We uncovered that SOD2-silenced hPMVECs exhibited significantly increased albumin leakage (Figure 4A). Similarly, transendothelial electrical resistance measured by ECIS was decreased in SOD2 knockdown cells, indicating barrier disruption (Figure 4B, Supplementary Figure S3A). To ascertain whether SOD2 mediated barrier disruption was due to diminished H_2_O_2_, siSOD2 hPMVECs were treated with H_2_O_2_. Decreased resistance was corrected with the addition of 4 μM H_2_O_2_ (Figure 4C, Supplementary Figure S3B), moreover barrier disruption from SOD2 knockdown was further exacerbated by the addition of PEG catalase (Figure 4D) suggesting a role for H_2_O_2_ signaling in regulating changes in SOD2 mediated permeability pathways. Sickle cell disease is characterized by episodes of increased hemolysis, which elevates levels of free hemoglobin and heme in the blood.^32^ Challenging sickle mice with extracellular hemin, the oxidized form of heme, triggers acute chest syndrome, a pulmonary complication of SCD characterized by increased permeability of the lung vasculature.^33^ We investigated whether simulating a hemolytic crisis by challenging siSOD2 hPMVECs with hemin would exacerbate differences in permeability. However, challenging siSOD2 hPMVECs with hemin did not further exacerbate the differences observed in permeability (Supplementary Figure S3C–F). These findings suggest that SOD2 maintains the endothelial barrier through H_2_O_2_ signaling in a hemin-independent pathway.

Another critical cellular function of the endothelium is the ability to proliferate and migrate during wound healing. One complication of SCD is the development of intractable and slow healing skin ulcers,^34^ indicating that there may be dysfunction in the endothelium's ability to proliferate and migrate. The scratch assay was used to measure wound closure in siSOD2 hPMVECs, and was found to be approximately half of siNT hPMVECs (Figure 4E and F), suggesting SOD2 is required for endothelial proliferation and migration. These data demonstrate that SOD2 is essential in the maintenance of multiple endothelial cell functions.

### SOD2 Mediates Adhesion and Integrin Protein Regulation in hPMVECs

**Figure 5.**
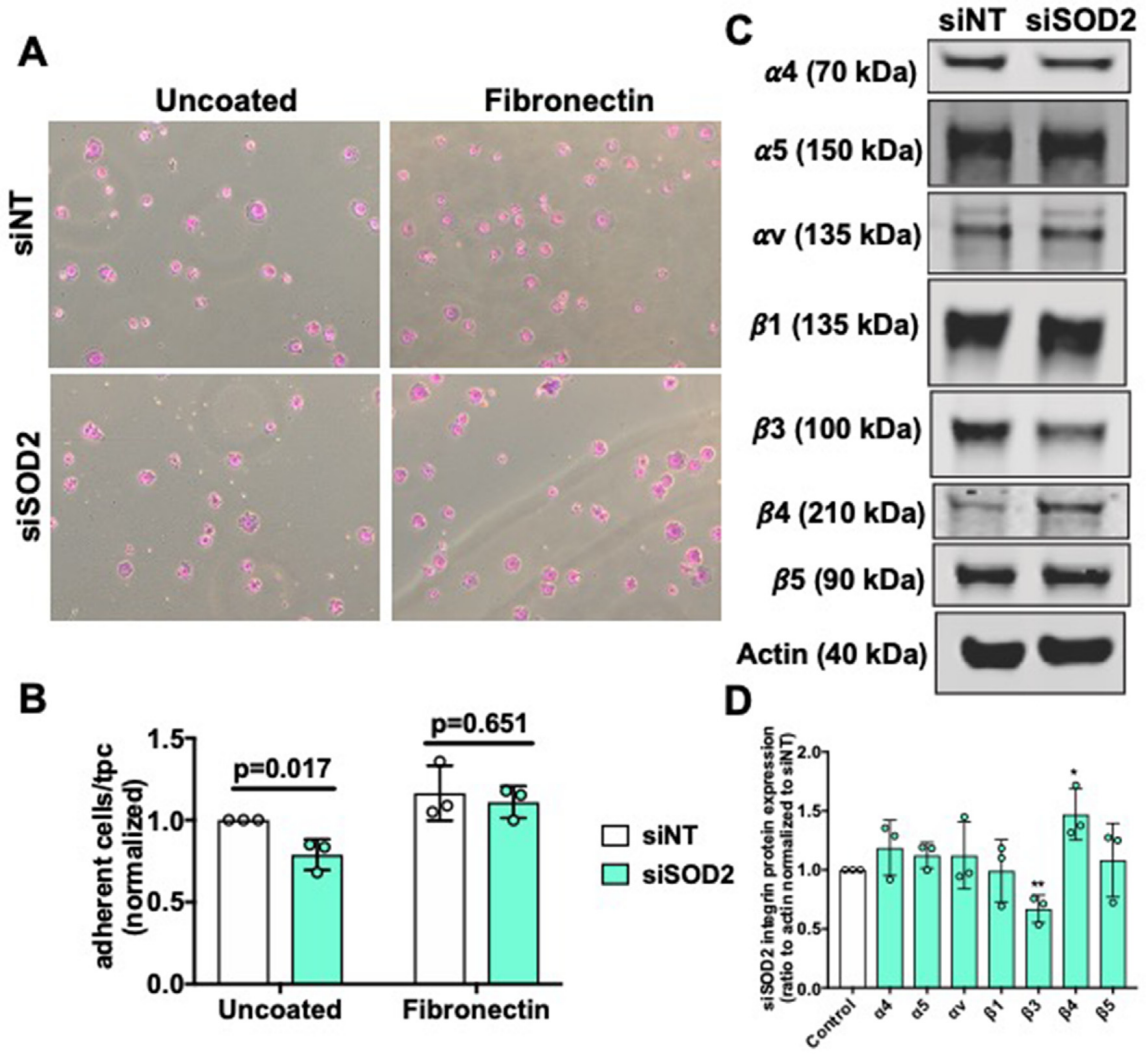
Decreased adhesion and integrin protein dysregulation in siSOD2 hPMVECs. (A) Representative images show attached viable siNT or siSOD2 hPMVECs in uncoated plates and plates coated with fibronectin. (B) Graphs demonstrate the number of cells per high power field (n = 3). (C) Western blot image of integrins α4, α5, αV, β1, β3, β4, β5, and actin protein expression. (D) Shows quantification of Western blot above (n = 3). Data shown represent averaged values for each day of experimentation with means ± SD. *P*-values determined by two-tailed Student's *t* test.

The assembly and turn-over of focal adhesions play essential roles in endothelial barrier integrity, proliferation, and migration.^35^, ^36^ Therefore, we examined whether SOD2 affects endothelial cell adhesion. First, adhesion of siSOD2 hPMVECs to uncoated cell culture plates was measured and was found to be decreased by 20% (Figure 5A and B). Integrins are essential mediators of cell-matrix adhesion.^37^ Interestingly, siSOD2 hPMVECs showed a significant decrease in integrin β3 protein expression and a significant increase in integrin β4 protein expression (Figure 5C and D). Given that integrin β3 mediates cell adhesion to fibronectin^38^ and that the initial stage of cell attachment is mediated by integrins^39^ we hypothesized that precoating cell culture plates with fibronectin would exacerbate differences observed in adhesion (Supplemental Figure S4). Instead of observing a greater difference in adhesion, precoating plates with fibronectin completely corrected the defect in adhesion (Figure 5A and B). Collectively, these results show that silencing of SOD2 disrupts cellular adhesion as well as diminishes integrin β3 protein expression and that pre-coating cell culture plates with fibronectin reverses the defect in adhesion, highlighting a potential disruption in siSOD2 hPMVECs produced fibronectin.

### Fibronectin Processing and Trafficking is Mediated by SOD2

**Figure 6.**
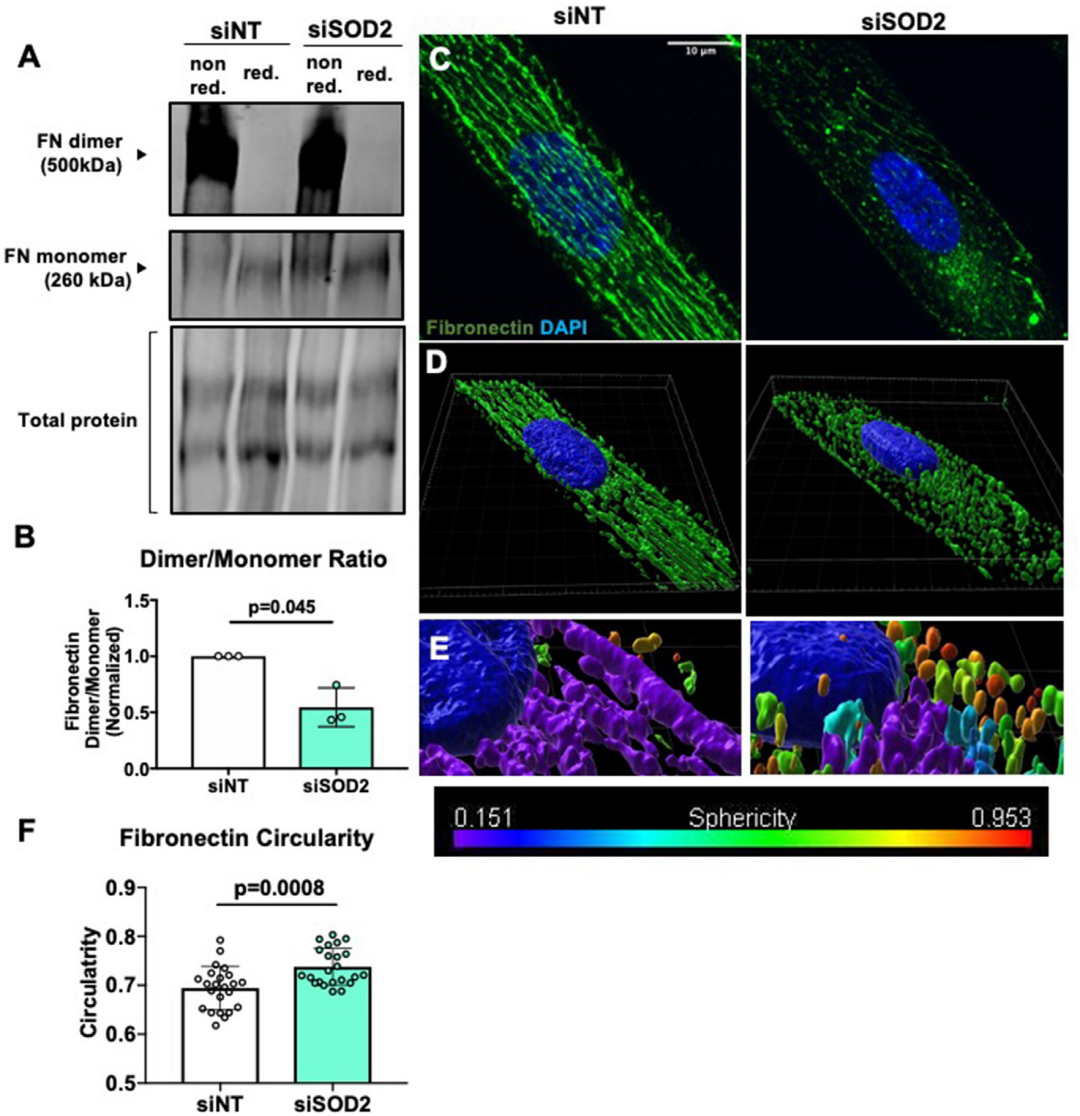
SOD2 knockdown disrupts fibronectin processing and trafficking. (A) Shows a Western blot image of nonreduced and reduced fibronectin and total protein. (B) Quantification of dimerized to monomeric fibronectin from above image (n = 3). (C) siNT or siSOD2 hPMVECs were plated onto PDRS coverslips stamped with lines of fibronectin and then stained for fibronectin in green and nuclei in blue. (D) 3D rendering of confocal images showing fibronectin distribution and organization. (E) 3D rendering of confocal images showing degree of spherecity of fibronectin, 0 indicating a perfect line and 1 indicating a perfect sphere (F) Quantification of fibronectin circularity (n = 23). Data shown represent values with means ± SD. *P*-values determined by two-tailed Student's *t* test.

Considering that adhesion deficiency in SOD2-silenced endothelial cells was corrected upon fibronectin addition, we hypothesized that SOD2 might be involved in fibronectin synthesis and assembly. No changes in *fibronectin* mRNA expression or total fibronectin protein expression were observed between siNT and siSOD2 hPMVECs (Supplementary Figure S5A–C). However, there was a significant decrease in the dimer to monomer ratio in siSOD2 hPMVECs (Figure 6A and B), indicating that there is reduced assembly of dimerized fibronectin, an essential step in the formation of fibronectin fibrils and integration into the extracellular matrix (ECM).^40^ Meanwhile, confocal microscopy showed a reduction in the formation of fibronectin bundles seen in siNT hPMVECs (Figure 6C). 3D rendering of the confocal images revealed that in SOD2 deficient hPMVECs, fibronectin is arrested within the cell (Figure 6D). Measurement of fibronectin circularity and sphericity showed that fibronectin within SOD2 KD hPMVECs was more globular as opposed to linear (Figure 6E and F). These results show that SOD2 plays a role in fibronectin matrix assembly.

## Discussion

Decreased SOD2 expression has been characterized in various disease states such as SCD,^26^ pulmonary hypertension,^21^ and chronic kidney disease.^41^ Given the evidence that therapeutic upregulation of SOD activity attenuates vascular dysfunction,^42^, ^43^ we hypothesized that SOD2 expression may be essential in modulating disease pathogenesis in conditions in which the vasculature is a primary target such as SCD. While it has been well established that there is an increase in oxidative stress in SCD with dysregulation of both enzymatic and non-enzymatic antioxidants,^4–6^, ^23^ there is no evidence demonstrating organ downregulation of antioxidants. Our study shows for the first time that there is significant downregulation in both endothelial and SMC SOD2 protein expression in the lung tissue of SCD patients. High flow oxygen, which is currently under clinical trial for treatment of vaso-occlusive pain crisis in SCD patients,^44^ has been shown to increase SOD2 activity in macrophages.^45^ Unfortunately, we are unaware if hyperoxic treatment was used by the patients in this study. We have also shown that this decrease in SOD2 protein expression is recapitulated in the Townes transgenic mouse model of SCD in whole lung tissue. These findings established the premise for further studies investigating the functional effect of decreased endothelial expression of SOD2.

We silenced SOD2 protein expression in hPMVECs and examined mitochondrial function. The significant increase in mitochondrial superoxide production with silencing of SOD2 (Figure 3A) demonstrated an essential role of SOD2 in modulating ROS levels within the mitochondria. Regardless of a 50% increase in mitochondria superoxide production, there were no deleterious effects on respiratory complex activity, mitochondrial respiration, or ATP production (Figure 3D–F) despite other studies having shown that OCR is decreased in SOD2 null HEK293T cells^46^ and chondrocytes.^47^ The preservation of oxygen consumption could be attributed to the limited time of exposure of the respiratory chain to increased superoxide or perhaps other mechanisms are able to partly compensate when there is increased mitochondrial oxidative stress. We did, however, observe a decrease in mitochondrial membrane potential (Figure 3G), which would imply the presence of mitochondrial damage.^48^ Given that this impairment in mitochondrial transmembrane potential formation was not significant enough to decrease ATP production, there may be other pathways, such as glycolysis, that are able to compensate for the lack of SOD2 to maintain ATP production.

Although the effects on mitochondria function were moderate, we observed significant endothelial dysfunction. Both albumin flux and ECIS showed that knockdown of SOD2 in hPMVECs increased endothelial permeability at baseline (Figure 4A and B, Supplemental Figure S3A). As far as we know, this is the first study to demonstrate that SOD2 is required to maintain endothelial barrier integrity. The impaired endothelial barrier function was reversed by H_2_O_2_ (Figure 4C, Supplemental Figure S3B). It has been well established that H_2_O_2_-mediated oxidation of cysteine residues is a crucial part of intracellular signaling.^49^ The reversibility of impaired endothelial barrier function with H_2_O_2_ demonstrates that redox signaling, possibly through the modification of oxidizable amino acid residues such as cysteine, is implicit in SOD2-mediated permeability regulatory pathways. Plasma heme levels are elevated in SCD.^50^ Hemin, the oxidized form of heme, is a known disruptor of endothelial barriers and has been implicated in the progression of SCD clinical complications such as acute chest syndrome.^33^, ^51^, ^52^ We investigated whether SOD2 knockdown exacerbates hemin-induced permeability. We found no difference with hemin treatment in siSOD2 hPMVECs compared to siNT hPMVECs (Supplemental Figure S3C–E). This result suggests that hemolytic products may be the cause of SOD2 downregulation in SCD as opposed to a potentiator of the effects of SOD2 down-regulation. One limitation of this study is the inability to monitor the effects of chronic stimulation with hemin, which would more closely model chronic hemolysis in SCD. In addition to increased permeability, endothelial migration and proliferation, measured through the *in vitro* wound healing assay, were also reduced by SOD2 siRNA (Figure 4D and E). Smooth muscle cell mitochondria relocate to the leading edge of the cell to facilitate mobility and migration.^53^ SOD2 may modulate cell migration and proliferation by facilitating H_2_O_2_ production at the leading edge. Future studies examining the spatial and temporal control of H_2_O_2_ production via SOD2 regulation are warranted. It is important to note that our study did not directly measure intracellular H_2_O_2_. This is limited by the lack of a specific intracellular H_2_O_2_ probe that is easy to deliver to primary endothelial cells. The functional effects of reduced SOD2 in pulmonary arterial SMC is also a future area of investigation.

Permeabiltiy, proliferation, and migration are all cellular functions reliant on sufficient integrity of focal adhesions.^54^, ^55^ We have shown that SOD2 deficient hPMVECs have reduced adhesion to uncoated cell culture plates (Figure 5A and B). Superoxide dismutase 2 depleted hPMVECs also had reduced integrin β3 protein expression (Figure 5C and D). Considering that integrin β3 facilitates adhesion to fibronectin,^38^ we expected that precoating cell culture plates with fibronectin would increase adhesion differences. Surprisingly, fibronectin precoating corrected adhesion deficiency in SOD2 knockdown cells (Figure 5A and B). This would imply that the defect in focal adhesion was due to a disturbance with cell-produced fibronectin and that decreased integrin β3 protein expression was most likely a result of an issue with fibronectin. Indeed, siSOD2 hPMVECs had a reduction in the ratio of dimerized to monomeric fibronectin (Figure 6A and B), suggesting that SOD2 facilitates fibronectin dimerization. This disruption in fibronectin processing was visually appreciable (Figure 6C). Upon performing 3D rendering of the images, we observed that SOD2 is not only necessary for fibronectin dimerization but also needed for proper localization to the outside of the cell (Figure 6D). Further analyses revealed that fibronectin in siSOD2 hPMVECs was more circular (Figure 6E and F). This measurement further supports the finding that SOD2 is needed for the dimerization and thereby the polymerization of fibronectin. Fibronectin has multiple domains to facilitate interactions with other ECM proteins, the cell, and other fibronectin proteins.^40^ Dimerized fibronectin is assembled intracellularly through oxidation of cysteine residues in the C110 region on the carboxy terminus.^56^, ^57^ Upon secretion and binding to cell surface receptors, dimers interact through disulfide bonds to cross-link into multimers.^56^ Ablation of these C-terminal cysteine residues results in complete failure to dimerize, though the resultant monomeric fibronectin can still be secreted.^57^ Lysyl oxidase has been identified as a catalyst for covalent cross-linking in fibrillar collagen as well as elastin.^58^ Lysyl oxidase has also been shown to bind with a strong affinity to C-terminal fragments of cellular fibronectin, suggesting that it may catalyze fibronectin dimer formation.^58^ No other peroxidases or oxidases have been implicated in directly or indirectly modulating fibronectin dimerization. Given the fact that mitochondria relocate to the leading edge of cells to facilitate migration^53^ and that fibronectin fibrillogenesis, which is reliant on fibronectin dimerization, is essential for promoting directionality in cellular migration,^59^ it is probable that H_2_O_2_ signaling from the mitochondria affects fibronectin processing. We propose that decreased H_2_O_2_ formation through knockdown of SOD2 destabilizes fibronectin dimers by decreasing oxidation of cysteine residues, thereby disrupting cellular migration. Failure of fibronectin dimerization would also decrease cellular adhesion^40^ and disrupt endothelial barrier function.^54^ Future studies are needed to verify whether mitochondria interact with fibronectin during dimerization and if impaired dimerization leads to physiological and pathophysiological consequences *in vivo* and in SCD.

In conclusion, we have shown for the first time that there is a decrease in endothelial cell SOD2 expression in SCD and that reduction in endothelial SOD2 protein expression results in disruption of fibronectin dimerization and secretion. This disruption of fibronectin processing consequently leads to decreased adhesion, migration, and proliferation, integrin protein dysregulation, as well as an increase in permeability. Taken together, these data suggest that further investigation into therapeutic treatments targeting SOD2-mediated changes in ECM protein assembly may be beneficial in SCD.

## Data Availibility

All data are available within this article and the online supplementary material.

## Supplementary Material

zqac005_Supplemental_Files

## Coflicts of Interest

The authors have no conflicts of interst to declare.
